# Immunohistochemical visualization of lymphatic vessels in human dura mater: methodological perspectives

**DOI:** 10.1186/s12987-023-00426-3

**Published:** 2023-03-28

**Authors:** César Luis Vera Quesada, Shreyas Balachandra Rao, Reidun Torp, Per Kristian Eide

**Affiliations:** 1 Department of Neurosurgery, Oslo University Hospital-Rikshospitalet, PB 4950 Nydalen, Oslo, 0424 Norway; 2 Institute of Clinical Medicine, Faculty of Medicine, University of Oslo, Oslo, Norway; 3 Division of Anatomy, Department of Molecular Medicine, Institute of Basic Medical Sciences, University of Oslo, Oslo, Norway

**Keywords:** Cerebral meninges, Human dura mater, Meningeal lymphatic vessels, Neuro-immunology

## Abstract

**Background:**

Despite greatly renewed interest concerning meningeal lymphatic function over recent years, the lymphatic structures of human dura mater have been less characterized. The available information derives exclusively from autopsy specimens. This study addressed methodological aspects of immunohistochemistry for visualization and characterization of lymphatic vessels in the dura of patients.

**Methods:**

Dura biopsies were obtained from the right frontal region of the patients with idiopathic normal pressure hydrocephalus (iNPH) who underwent shunt surgery as part of treatment. The dura specimens were prepared using three different methods: Paraformaldehyde (PFA) 4% (Method #1), paraformaldehyde (PFA) 0.5% (Method #2), and freeze-fixation (Method #3). They were further examined with immunohistochemistry using the lymphatic cell marker lymphatic vessel endothelial hyaluronan receptor 1 (LYVE-1), and as validation marker we used podoplanin (PDPN).

**Results:**

The study included 30 iNPH patients who underwent shunt surgery. The dura specimens were obtained average 16.1 ± 4.5 mm lateral to the superior sagittal sinus in the right frontal region (about 12 cm posterior to glabella). While lymphatic structures were seen in 0/7 patients using Method #1, it was found in 4/6 subjects (67%) with Method #2, while in 16/17 subjects (94%) using Method #3. To this end, we characterized three types of meningeal lymphatic vessels: (1) Lymphatic vessels in intimate contact with blood vessels. (2) Lymphatic vessels without nearby blood vessels. (3) Clusters of LYVE-1-expressing cells interspersed with blood vessels. In general, highest density of lymphatic vessels were observed towards the arachnoid membrane rather than towards the skull.

**Conclusions:**

The visualization of meningeal lymphatic vessels in humans seems to be highly sensitive to the tissue processing method. Our observations disclosed most abundant lymphatic vessels towards the arachnoid membrane, and were seen either in close association with blood vessels or remote from blood vessels.

**Supplementary Information:**

The online version contains supplementary material available at 10.1186/s12987-023-00426-3.

## Introduction

The study of meningeal lymphatic vessels has a long history. In 1787, the Italian anatomist Giovanni Paolo Mascagni for the first time demonstrated lymphatic vessels within the human dura, which he described in a Latin text termed *Vasorum Lymphaticorum Corporis Humani Historia et Ichnographia* (English *History and Graphical Representation of the Lymphatic Vessels in the Human Body*) [1]. While this text was forgotten for many years, the study of meningeal lymphatic vessels later attracted the interest of several authors. Lecco described lymphatic structures within the human dura [2], and Földi studied lymphatic structures within dura mater of dogs [3], as well as functional consequences of cervical lymphatic blockade in dogs, cats and rats [4]. Furthermore, Andres et al. [5] showed lymphatic structures in dura of rats. Cserr explored the role of lymphatic drainage of macromolecules from the brain, as well as its role in immune surveillance [6–8]. More recently, lymphatic structures were identified in the dural portion of the optic nerve in autopsy specimens [9, 10], and in the murine craniofacial region [11]. Moreover, Johnston studied lymphatic drainage of cerebrospinal fluid (CSF) via the cribriform plate, particularly addressing its possible role in hydrocephalus [12–16].

In 2015, the study of meningeal lymphatic vessels gained greatly renewed interest, when functional lymphatic vessels within the dura mater of rodents were reported [17, 18]. These studies demonstrated lymphatic vessels nearby the dural sinus veins of rodents that were able to carry fluid and immune cells from the CSF [17]. Moreover, loss of dural meningeal vessels abrogated the transport of macromolecules to the deep cervical lymph nodes [18]. Later, a growing body of evidence points at a pivotal role of the meningeal lymphatic vessels for egress of waste solutes from the central nervous system (CNS), as well as their role in immune surveillance of the CNS [19–22]. However, the existing evidence about meningeal lymphatic function largely derives from animal studies, while human data exclusively rely on observations in autopsy specimens [23–28]. Given the possible great importance of meningeal lymphatic structures for normal function of the CNS as well as diseases of the CNS, there is an urgent need for establishing methodology to characterize meningeal lymphatic vessels in humans.

This present study was undertaken to examine methodology for immunohistochemistry of human dural lymphatic vessels. To this end, we examined dura mater of patients with the dementia subtype idiopathic normal pressure hydrocephalus (iNPH) who as part of treatment underwent shunt surgery. Dura mater was retrieved from the same location in all subjects, and we compared three different fixation methods in order to characterize the meningeal lymphatic vessels in humans. We decided to examine dura mater in iNPH patients since these patients present with delayed molecular clearance from subarachnoid CSF [29], impaired glymphatic function [30, 31] as well as altered CSF to blood clearance [32]. Given the previous data that loss of dural meningeal vessels impairs the transport of macromolecules to the deep cervical lymph nodes [18], we hypothesize that meningeal lymphatic function could be affected in iNPH, and therefore of value to examine in this patient group. Perhaps a dural biopsy during shunt surgery may add diagnostic information to this disease? In order to determine the importance of dural lymphatics in disease such as iNPH, methods must be developed to assess these vessels in tissue biopsies of these patients.

## Methods

### Patients

We included patients with iNPH who underwent shunt surgery, and who followed the ordinary clinical routine for shunt surgery within the department of neurosurgery. Notably, it was beyond the scope of this study to compare meningeal lymphatic vessels in iNPH versus other diseases or control subjects.

### Tissue sampling and preparation

**Fig. 1:**
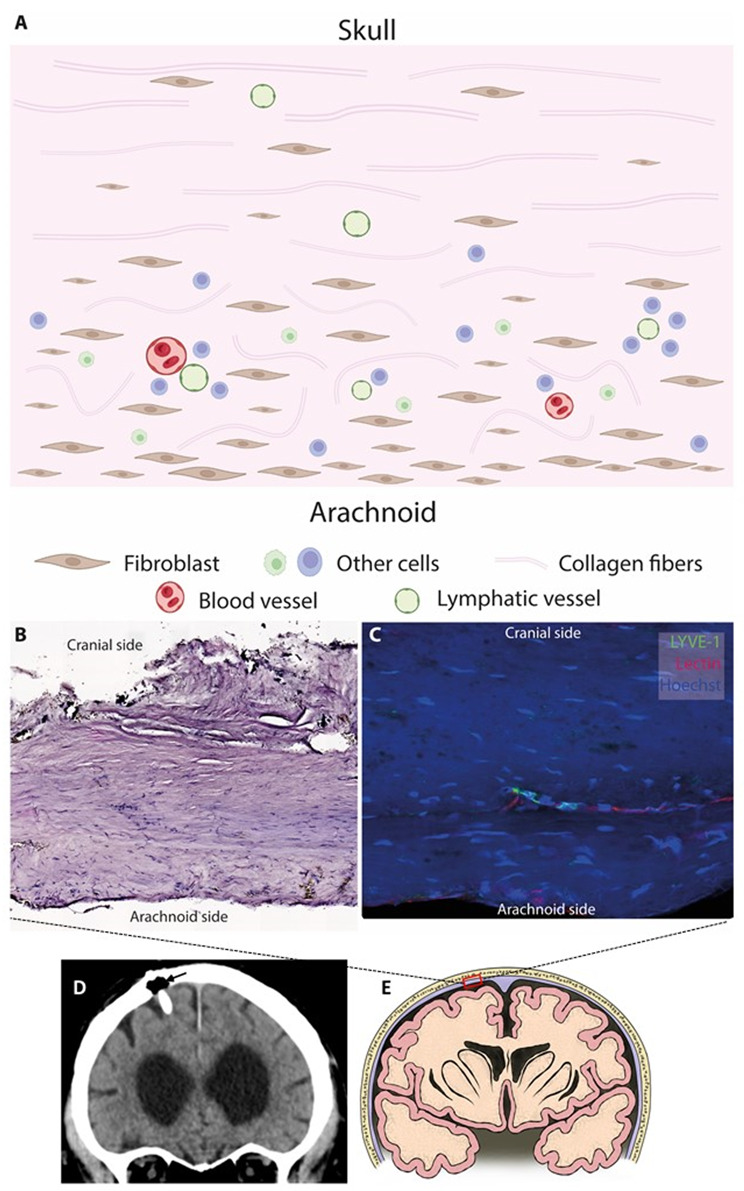
**Dura Mater schematic illustrating morphological features for orientation.** Collagen fiber is more uniformly oriented towards the skull and less uniform towards the arachnoid membrane (**A**). In addition, cellular density is low towards the skull with only a few fibroblasts present while the number of cells increases towards the arachnoid membrane. Bottom panels are representative images showing cellular density and collagen fiber orientation throughout the dura mater stained with cresyl violet (**B**) or through IHC (**C**). The dura biopsy was obtained from the site of shunt catheter insertion in all iNPH patients. (**D**) The trepanation (arrow) was placed in the right frontal region, about 12 cm posterior to glabella, and average 1.6 cm lateral to the superior sagittal sinus. (**E**) The dura (marked by red rectangle) was incised circumferential (diameter about 5 mm) in its entire thickness (about 1 mm) and removed in one piece. Illustration in **E**: Øystein Horgmo, University of Oslo

The dura mater was sampled during ventriculoperitoneal (VP) shunt surgery based on clinical routine at the department of neurosurgery in Oslo University Hospital – Rikshospitalet. The trepanation necessary for the surgical procedure and tissue sampling was performed 12 cm caudally from the glabella and 1–2 cm lateral to the midline (Fig. 1). The surgical procedure was performed using an operating microscope. A burr hole less than 1 cm in diameter was made and the underlying dura exposed. With a knife, the dura was incised circumferential (diameter about 5 mm) in its entire thickness (about 1 mm) and removed in one piece. Once extracted, the sample was prepared with one of three methods.

#### Method #1

Six dura samples (numbered #1 to #6) were immersed in 4% paraformaldehyde (PFA). The following day the samples were transferred for preservation to a diluted (0.4%) PFA solution. One day prior to sectioning, they were cryoprotected in graded sucrose solutions (10% for 30 min, 20% for one hour and 30% sucrose overnight in 0.1 M phosphate buffer). For sectioning the samples were frozen on dry ice in OCT medium (Richard-Allan Scientific™ Neg-50™, Thermo Fisher Scientific; Cat#6502) and sectioning was performed in a Cryostat NX70 from Thermo Scientific with vacutome. It was not possible to determine the rostro-caudal or medio-lateral orientation in the horizontal plane of the tissue. Therefore, the sections obtained were either sagittal or coronal and 16 μm thick. Cranial (outer) vs. arachnoid (inner) orientation in the vertical plane of the tissue was deciphered through microscopy (See “Microscopy and measurements”). These sections were immediately mounted on Superfrost™ Plus microscopy slides (Thermo Fisher Scientific) and stored at -20 °C until immunohistochemistry (IHC). This fixation method did not produce any staining (see Discussion) and therefore the experiments with these samples were repeated with antigen retrieval methods (described below). Antigen retrieval did not result in any significant improvement and this lead to trying Method#2 with the subsequent patient samples.

#### Method #2

As with Method #1, seven dura samples (numbered #7 to #13) were immersed in 0.5% PFA immediately after resection and was fixed overnight at 4 °C. One day prior to sectioning, they were cryoprotected in graded sucrose solutions, as mentioned above. Sectioning was performed as described for Method #1. The thickness of the sections was modified to 30 μm to prevent washing off the glass slide, which was frequent with Method #1 particularly after antigen retrieval. These sections were immediately mounted on Superfrost™ Plus microscopy slides (Thermo Fisher Scientific) and stored at -20 °C until IHC. This method resulted in a small labelling improvement (see Results), however to improve even further we moved on to Method #3 with the rest of included patient samples.

#### Method #3

The remaining 17 dura samples (numbered #14 to #30) included in the study were immediately frozen on dry ice in OCT medium (Richard-Allan Scientific™ Neg-50™, Thermo Fisher Scientific; Cat#6502) after resection and stored at -80 °C until sectioning. For sectioning, the frozen samples were transferred to the cryostat machine. Sectioning was performed similar to Methods #1 and #2, but without the sucrose steps. The sections obtained were either sagittal or coronal with a thickness of 30 μm. These sections were immediately mounted on Superfrost™ Plus microscopy slides (Thermo Fisher Scientific) and stored at -80 °C until IHC. On the day of IHC the slides were taken out from the − 80 °C, allowed to sit at room temperature for 10 min and the sections were fixed on the slide with 0.5% PFA for 10 min.

### Immunohistochemistry

For immunolabeling of lymphatic endothelial cells (LECs) the sections were thawed at room temperature, rinsed 3 times for 5 min each in 0.01 M phosphate buffer saline (PBS). To reduce unspecific binding of antibodies the sections were blocked for 60 min with a blocking solution (10% normal donkey serum, 1% bovine serum albumin (BSA), 0.5% Triton X-100 in PBS). Afterwards they were incubated in blocking solution containing 0.01% sodium azide with the primary antibody against lymphatic vessel endothelial hyaluronan receptor 1 protein (LYVE-1) (polyclonal rabbit anti-LYVE1: Abcam cat#ab33682, RRID:AB_881387, 1:500 dilution) or podoplanin (PDPN) (GeneTex cat#50,043, RRID:AB_11166297, 1:1000 dilution) overnight at 4 °C. On the following day the sections were rinsed 3 times for 10 min each in 0.01 M PBS following incubation in the blocking solution with the secondary antibody (Cy3 donkey anti-rabbit; Jackson ImmunoResearch Labs; Cat#:711-165-152; RRID:AB_2307443, 1:250 dilution) for 30 min and another 30 min with the secondary antibody plus DyLight® 649 conjugated tomato lectin (LEL, TL; Vector labs; Cat#: DL-1178) to stain blood vessels. The sections were then rinsed again three times for 10 min each in 0.01 M PBS. Afterwards, Hoechst 33,258 was used for nuclear staining (Thermo Fisher Scientific; Cat#: H3569; RRID:AB_2651133, 1:5000 dilution) for five minutes. Lastly the sections were rinsed in distilled water, twice for five minutes, and coverslipped using ProLong™ Gold Antifade Mountant (Thermo Fisher Scientific; Cat#: P36934; RRID:SCR_015961).

Prior to using DyLight® 649 conjugated tomato lectin, mouse antibodies against CD31 were used as blood vasculature marker incubated in conjunction with the antibody against LYVE-1 and the next day incubated with Cy2 donkey anti-mouse secondary antibody. However we opted for continuation of lectin instead of CD31 (see Results section).

As negative controls, some sections were incubated in the same solution without the primary anti-LYVE-1/PDPN antibody. For positive controls, mouse liver and spleen samples of C57/BL6 genetic background were dissected immediately after cervical dislocation and frozen on dry ice. These mouse samples were prepared similarly to Method #3. Since liver is abundant in lymphatic vessels while the spleen lacks them, they serve adequately as controls for antibody specificity.

### Antigen Retrieval

The following procedures were followed for antigen retrieval.

- The sections were washed in 0.01 PBS 3 times for 5 min followed by immersion in 0.2 M Tris-HCl for 30 min at 95 °C. Afterwards, the IHC protocol described above was done as described.
- The IHC protocol described above was done as described; however, all solutions were prepared with 1% Triton X-100 instead of 0.5%.
- The sections were washed in 0.01 PBS with Tween20 (PBST) 2 times for 5 min followed by application of 80% formic acid to each section on the glass slide for 2 min at 95 °C. Afterwards, the sections were rinsed in PBST 3 for 5 min and the IHC protocol described above was done as described.
- The sections were washed in 0.01 PBS 3 times for 5 min followed by immersion in 1 mM EDTA for 30 min at 90 °C. Afterwards, the IHC protocol described above was done as described.
- For sodium citrate buffer with heat induced antigen retrieval, the sections were washed in 0.01 PBS 3 times for 5 min followed by rinsing in distilled water. These were then immersed 0.1 M sodium citrate buffer pH: 6.0 and microwaved for 2:30 min at 700 W plus 5 min at 350 W. Sections were allowed to cool to room temperature rinsed again in distilled water and then blocked and incubated with primary antibody as described above.

### Microscopy and measurements

High-resolution images were acquired using a LSM 710/700 confocal microscope (Carl Zeiss Microscopy) at magnification of 20x or 63x w/oil immersion objective. To compare the tissues processed with different fixation methods, the images were acquired using the same settings. They were processed using the Zen Blue software (Carl Zeiss Microscopy). In some instances for 3D visualization, a Z-stack image was acquire with a slice thickness between 13 and 19 μm depending on the thickness of the dura sample. The measurement of lymphatic vessels was performed using the same software in the sections were most surface area was visually labelled (see limitations in Discussion).

To distinguish between cranial and arachnoid side of the dura mater, overall collagen appearance and cellular density was used (Fig. 1). Towards the cranial side of the dura, collagen fibers are oriented more uniformly with a low density of fibroblasts and cells while the arachnoid side exhibits less organized collagen fibers due to the high density of cells and structures like vessels [33–35].

### Statistics

The statistical analyses were performed using SPSS version 27 (IBM Corporation, Armonk, NY, USA). Differences between groups were determined by analysis of variance (ANOVA) with post-hoc Bonferroni corrections for continuous variables and the Chi-square test for categorical variables. Two-tailed p-values of less than 5% were accepted as statistically significant.

## Results

### Patients

Thirty iNPH patients who underwent shunt surgery were included in the study. Demographic data are shown in Table 1. The dura biopsy, sized about 5 mm, was obtained from the same place as the ventricular catheter was inserted during shunt surgery, on average 16.1 ± 4.5 mm lateral to the superior sagittal sinus (Table 1). Although different fixation protocols for dura mater were used, patients did not differ significantly concerning age or gender group, and distance from superior sagittal sinus was not different between groups. The biopsy was not accompanied with adverse events.

**Table 1:** Patient material

	Total		Fixation method			Significance
			Method #1 (PFA 4.0%)	Method #2 (PFA 0.5%)	Method #3 (Freeze)	
***Demographic***						
N	30		7	6	17	
Age (yrs)	73.1 ± 6.5		72.7 ± 7.4	73.7 ± 8.9	73.1 ± 5.6	ns
Gender (f/m)	12/18		2/5	2/4	8/9	ns
***Dura localization***						
Distance from SSS* (mm)	16.1 ± 4.5		19.0 ± 5.0	13.5 ± 4.0	15.9 ± 3.9	ns

*SSS: Superior sagittal sinus. Ns: Non-significant. Analysis of variance (ANOVA) with post-hoc correct tests for continuous data and Pearson Chi square test for categorical data

### Impact of tissue processing and preparation

**Fig. 2:**
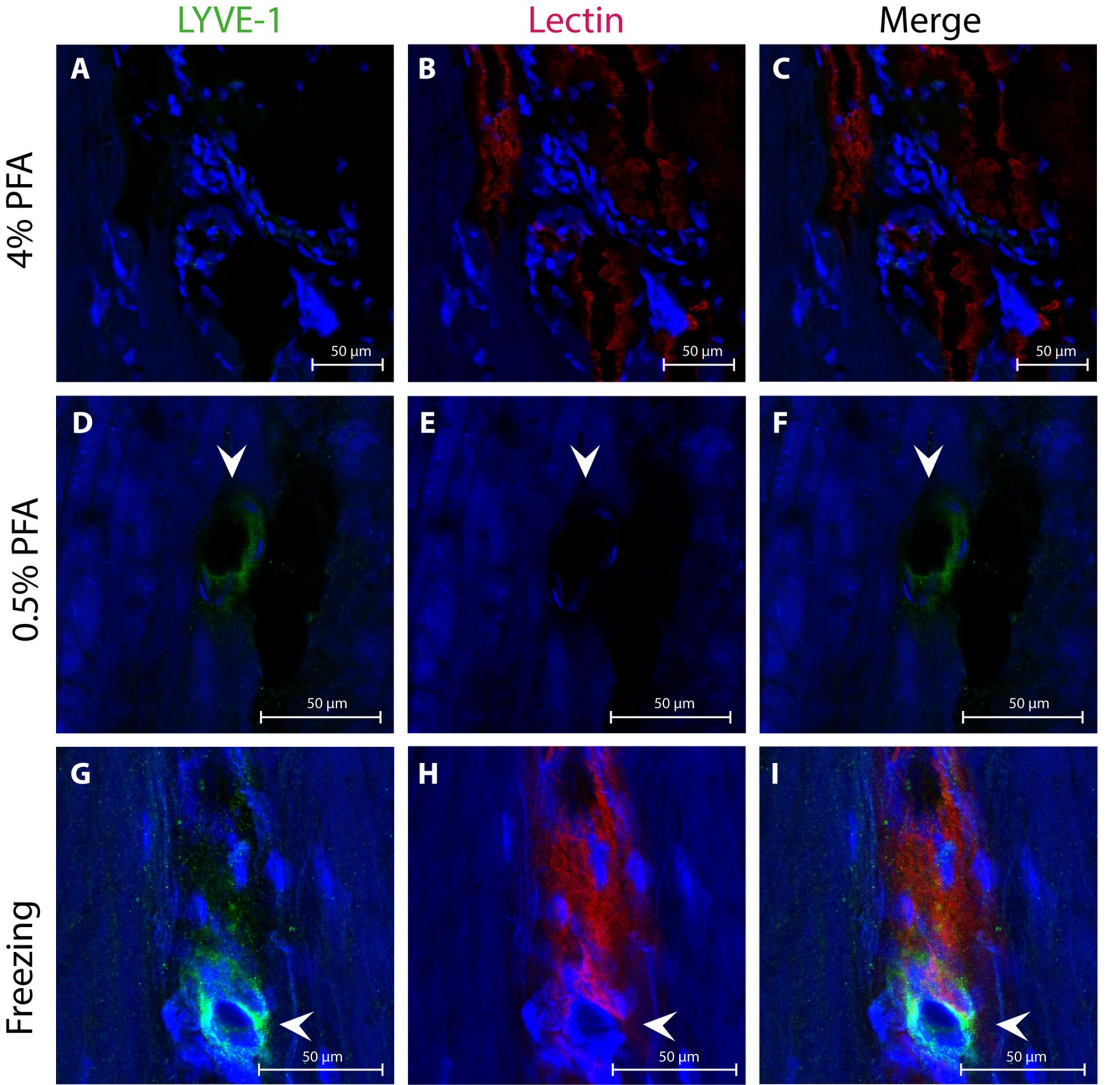
**Comparison between the three different method groups.** Top (**A-C, patient #5**) middle (**D-F**, patient #28) and bottom rows (**G-I**, patient #15) show representative sections of the different groups with fixations of Methods #1, #2 and #3, respectively. In the left column, LYVE-1 staining produced a distinctly stronger signal when comparing Method #2 to Method #3 while is virtually absent with Method #1

In dura specimens fixated with PFA 4% (Method #1), we found no evidence of lymphatic structures despite trying several antigen retrieval methods (Table 2). Reducing the strength of fixative to PFA 0.5% (Method #2) enabled visualization of lymphatic structures in 4/6 (67%) individuals; lymphatic vessels together with blood vessels in 1/6 (17%), lymphatic vessels remote from blood vessels in 2/6 (33%) and cluster-like lymphatic structures in 1/6 (17%) subjects (Table 2). On the other hand, fixation with the freezing method (Method #3) demonstrated lymphatic structures in 16/17 (94%) subjects, with lymphatic vessels together with blood vessels in 10/17 (59%), lymphatic vessels remote from blood vessels in 10/17 (59%) and cluster-like lymphatic structures in 7/17 (41%) subjects (Table 2). All the images were acquired using the same microscopic settings for comparing the different fixation methods. Hence, the fixation protocol is crucial for the identification of meningeal lymphatic vessels (Fig. 2). The results produced by different immunohistochemistry protocols for lymphatic vessels for Methods #1–3 are further illustrated in Supplementary Fig. 1. Method #3, however, also produced an apparently weaker blood vessel staining that led us to use lectin instead of CD31 due to simplicity during IHC.

**Table 2:** Immunohistochemical visualization of dural lymphatic structures depending on fixation method

Lymphatic structures	Fixation method		
	Method #1 (PFA 4.0%)	Method #2 (PFA 0.5%)	Method #3 (Freeze)
***Absence of lymphatic structures (n; %)***	7/7 (100%)	2/6 (33%)	1/17 (6%)
***Presence of lymphatic structures (n; %)***	0/ (0%)	4/6 (67%)	16/17 (94%)
Lymphatic vessels with blood vessels (n; %)	-	1/6 (17%)	10/17 (59%)
Lymphatic vessels without blood vessels (n; %)	-	2/6 (33%)	10/17 (59%)
Clusters of LYVE-1-expressing cells (n; %)	-	1/6 (17%)	7/17 (41%)

### Immunohistochemistry of meningeal lymphatic vessels

**Fig. 3:**
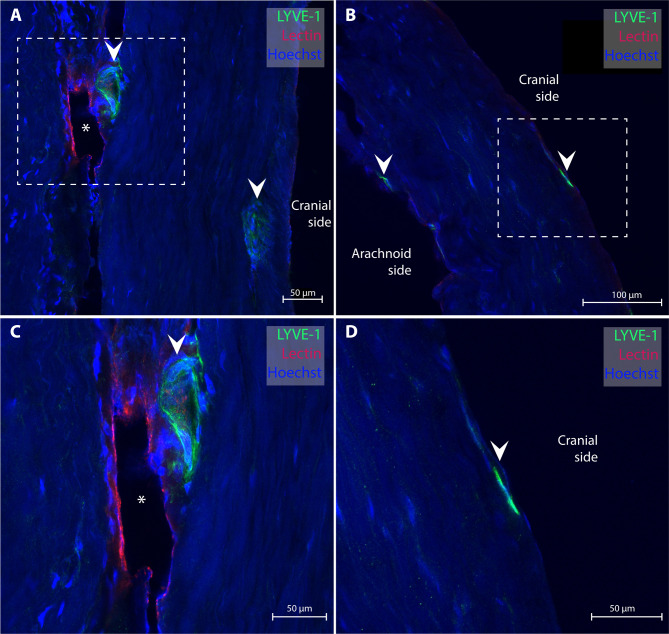
**Meningeal lymphatic vessels are most abundant towards the arachnoid membrane.** Overview section of dura showing that lymphatic vessels (white arrowheads) are found predominantly towards the inner membrane facing the arachnoid. We observed lymphatic vessels that (**A**) are close to blood vessels (asterisk), as well as (**B**) lymphatic vessels remote to blood vessels. In green LYVE-1 staining for visualizing of lymphatic endothelial cells, in red Lectin for endothelial blood vessels and in blue nuclear staining. In panels **C** and **D** magnification of **A** (patient #8) and **B** (patient #10), respectively

**Fig. 4:**
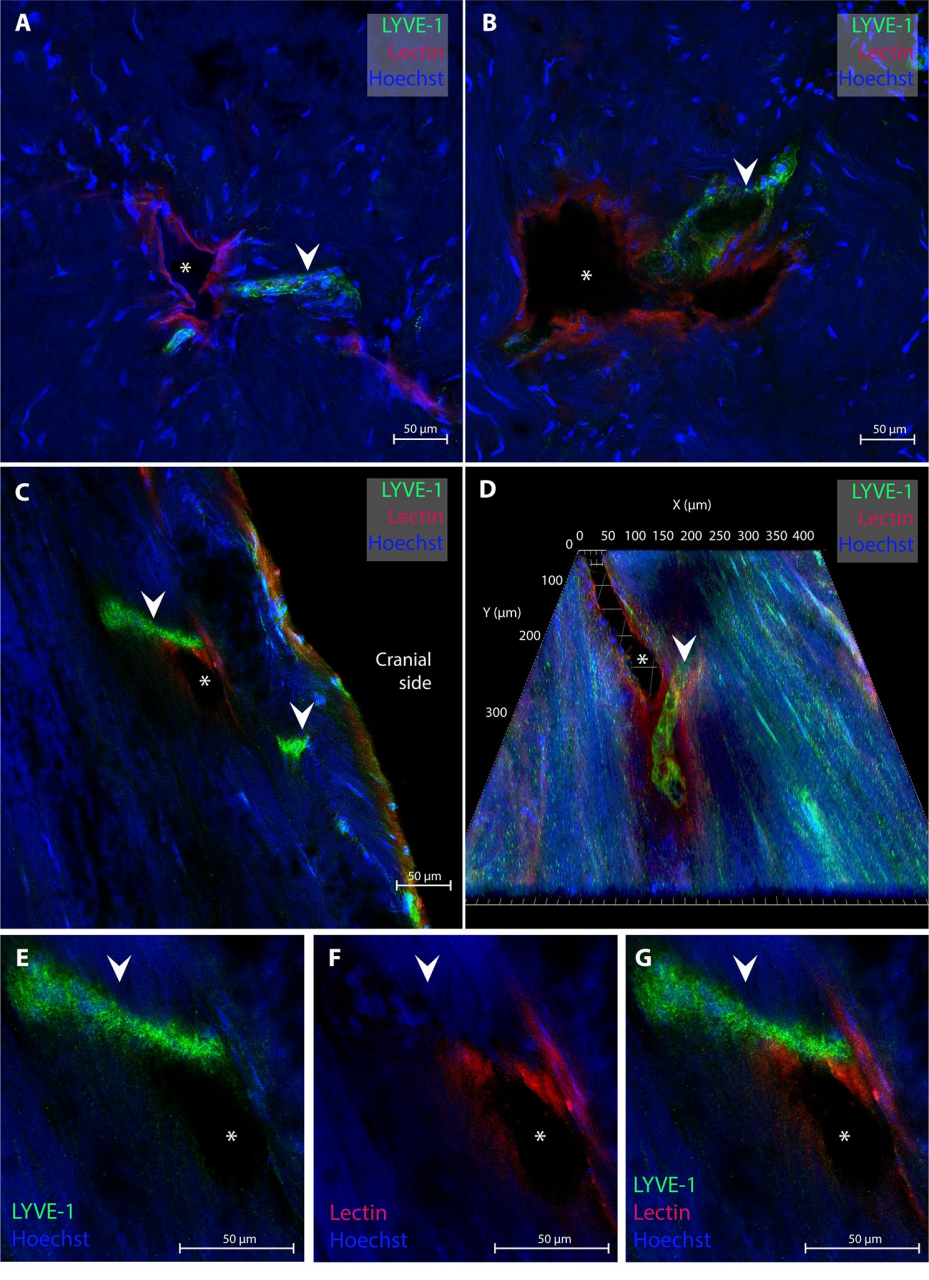
**Lymphatic vessels in close proximity to blood vessels.** **A-D** Examples of dural lymphatic vessels (white arrowheads), green color, that appear to travel on or close to blood vessels (asterisk). Section in panel **A** and **B** belong to patient #9; sections in panels **C** and **D** belong to patient #21. In **A**-**B**, note the accumulation of cells around both the lymphatic and blood vessel types. Panels **E-G** are a magnification of **C** with only the lymphatic staining (**E**), blood vessel staining (**F**) and both superimposed (**G**)

**Fig. 5:**
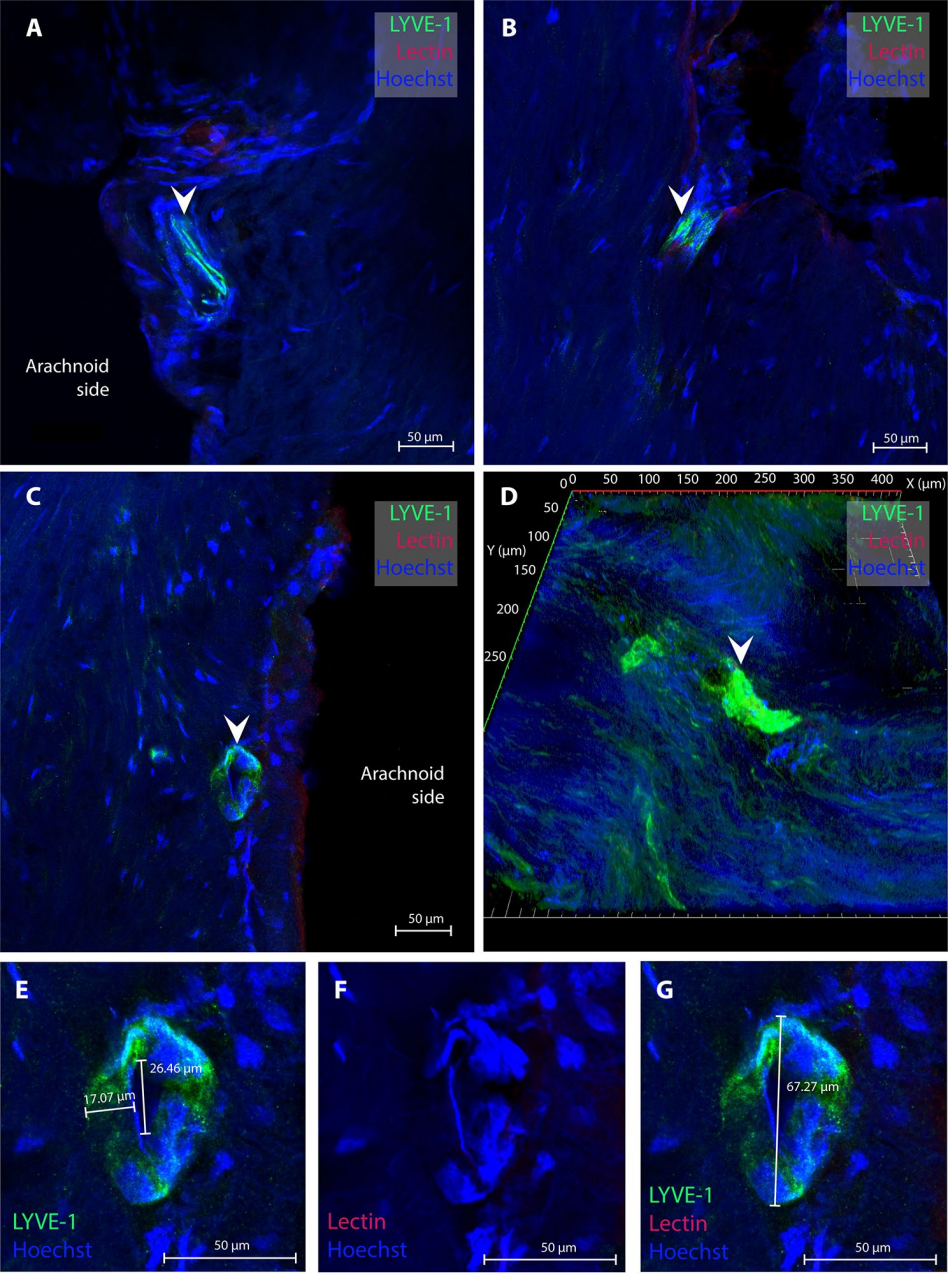
**Meningeal lymphatic vessels in distance from blood vessels.** **A-D** A characteristic observation was the presence of lymphatic vessels (white arrowheads) in distance from blood vessels, here illustrated by LYVE-1 positive vessels with no Lectin-stained blood vessels nearby. Section in panel **A** belongs to patient #8, section in panels **B** and **C** to patient #16 and section in panel **D** to patient #22. Panels **E-G** are a magnification of **C** with only the lymphatic staining (**E**), blood vessel staining (**F**) and both superimposed (**G**). Lastly, example measurements of lymphatic vessel lumen, wall thickness and overall width can be seen in **E** and **G**

**Fig. 6:**
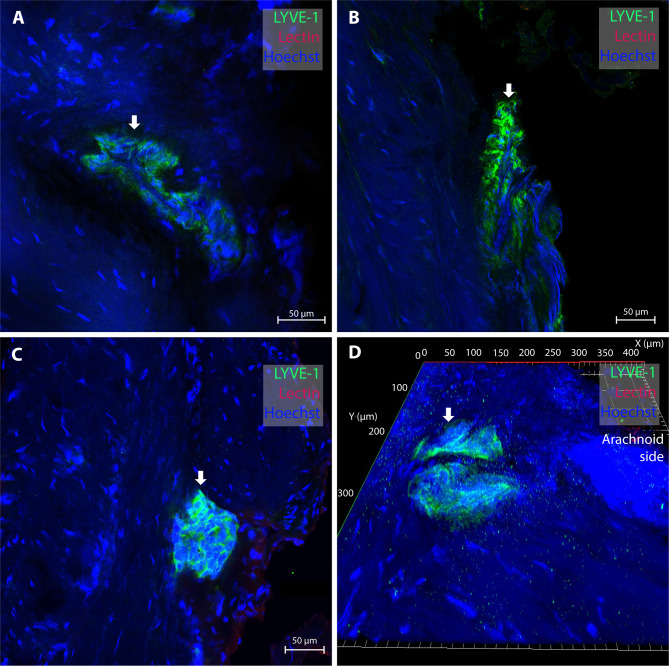
**Cluster of LYVE-1-expressing cells.** **A-D** We observed LYVE-1-expressing cells in a cluster-like fashion. Conglomerations of stained cell nuclei can be seen in all panels (white arrows). Sections in panels **A** and **D** belongs to patient #13, section in panel **B** belongs to patient #18 and section in panel **C** belongs to patient #10

The meningeal lymphatic vessels were found throughout the entire thickness of dura mater, but seem to be more predominant towards the arachnoid (Fig. 3). Meningeal lymphatic vessels were seen close to either blood vessels (Fig. 4; Supplementary Movie 1) or remote from blood vessels (Fig. 5; Supplementary Movie 2). There were no apparent differences between these lymphatic vessels. The lymphatic vessels nearby blood vessels had a wall thickness 5.3 ± 2.8 μm, lumen diameter 27.6 ± 12.7 μm, and total width of 44.3 ± 19.7 μm. Similarly, the lymphatic vessels remote from blood vessels had a wall thickness 7.2 ± 5.0 μm, lumen diameter 27.3 ± 17.6 μm, and total width of 48.0 ± 27.2 μm (Fig. 5E, G). Expression of LYVE-1 was also found in aggregated cell clusters (Fig. 6; Supplementary Movie 3), which we denote LYVE-1-expressing cells. These cell clusters had no apparent expression of vascular endothelial cells.

We tested the specificity of LYVE-1 staining, by ruling out unspecific binding from the fluorophore-conjugated secondary antibody and auto-fluorescence (see Methods) (Supplementary Fig. 2). To ensure the specificity of the antibody against LYVE-1, we tested for staining in mouse liver as positive control and mouse spleen as negative control (Supplementary Fig. 3). The liver exhibited pronounced staining for LYVE-1 antibodies due to the abundance of lymphatic vessels, while no staining was observed in spleen due to lack of lymphatic vessels, validating the specificity of the antibody.

**Fig. 7:**
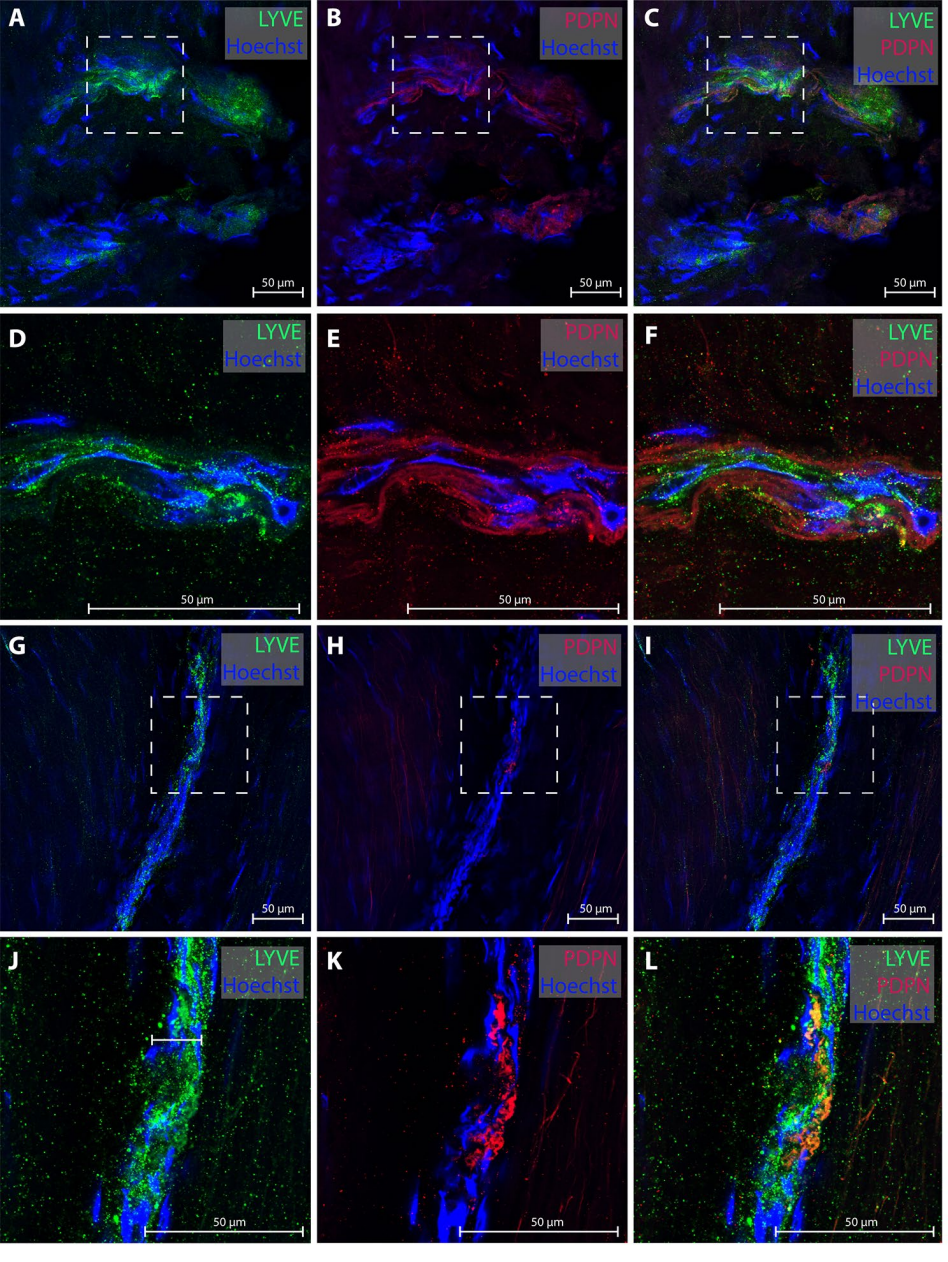
**Validation of LYVE-1 observations with PDPN.** Representative sections show images (patient #8, **A-F** and #15, **G-L**) where co-localized lymphatic markers PDPN and LYVE-1 confirmed the presence of meningeal lymphatic vessels. Left column panels show staining with LYVE-1, middle panels with PDPN and right column panels show overlap of LYVE-1 and PDPN. Panels **D-F** are a magnification of the area shown in **A-C** and panels **J-L** are a magnification of the area shown in **G-I**.

In order to validate the lymphatic identity of the LYVE-1 positive immunoreactive structures, we confirmed co-labeling with both markers LYVE-1 and PDPN in lymphatic vessels (Fig. 7). The control experiments of specificity of PDPN are shown in Supplementary Fig. 4.

Furthermore, we show that the LYVE-1 receptor is expressed in lymphatic endothelial cells but not vascular endothelial cells or red blood cells. In Supplementary Fig. 5, we show blood vessels where vascular endothelial cells have been labeled with Lectin, but also LYVE-1 labeling intramurally (tunica media), highlighting the importance of control-staining not to misidentify blood vessels as lymphatic vessels. The wall thickness was larger in blood vessels (Supplementary Fig. 5).

## Discussion

The present study demonstrates that successful immunohistochemistry of meningeal lymphatic vessels in human dura heavily relies on the fixation procedure. This suggests that the LYVE-1 receptor protein in meningeal lymphatic vessels is sensitive to both concentration and duration of fixation. The presently characterized meningeal lymphatic vessels were either located in close vicinity to blood vessels or remote from blood vessels, and we demonstrated LYVE-1-expressing cells gathered in clusters.

We primarily used the lymphatic cell marker lymphatic vessel endothelial hyaluronan receptor 1 (LYVE-1) for immunohistochemistry of meningeal lymphatic vessels. This is a type I integral membrane glycoprotein that binds to soluble and immobilized hyaluronan. To confirm the lymphatic identity of the LYVE-1 reactive structures, we used podoplanin (PDPN) [17, 18]. Other lymphatic markers include vascular endothelial growth factor 3 (VEGFR-3) and chemokine ligand 21 (CCL21). Utilizing these available markers of lymphatic endothelial cells, the vast majority of data is derived from experimental studies in animals [17, 18]. Kutomi and Takeda [36] studied cranial arachnoid granulations of dura in pigs and found the endothelial lining of granulations to be LYVE-1 immunoreactive, indicating they are lymphatic cells. These authors also referred to endothelial-like cell lined gaps in dura towards the subarachnoid space.

To the best of our knowledge, this is the first study to report the successful staining of meningeal lymphatic vessels in tissue biopsies from patients. It is, however, highly warranted to get experience with assessment of lymphatic vessels in dura mater of patients, given the marked species differences of the dura mater [37]. To this end, the available human data is derived exclusively from autopsy specimens though the number is low and the results somewhat variable. Louveau et al. [17] reported LYVE-1 staining of lymphatic vessels nearby the superior sagittal sinus in one case, and Absinta [23] reported in three autopsy specimens, dural lymphatic vessels immunoreactive to the antibodies against D2-40, PROX-1, CCL21 and LYVE-1. Visanji et al. [24] in four autopsy specimens visualized dural lymphatic vessels nearby the superior sagittal sinus, utilizing a monoclonal primary antibody to podoplanin, D2-40. The same antibody previously was shown to visualize lymphatic vessels of the dura part of the optic nerve [25]. Another study showed immunoreactive meningeal lymphatic vessels in ten autopsy specimens, with the help of the markers LYVE-1 and PDPN [26]. Moreover, Godman et al. [27] presented 21 autopsy specimens in whom 19/21 subjects showed positive staining for lymphatic vessels, using the antibodies against D2-40 and LYVE-1. These authors differentiated between two kinds of meningeal lymphatic vessels, namely lymphatic vessels comprising a single layer of endothelium with no smooth muscle, unoccupied lumen and LYVE-1 positive (Type 1) and another type of lymphatic vessels with material within the lumen being LYVE-1 negative. Type 1 lymphatic vessels were found in the periosteal and meningeal layers of the dura mater while Type 2 was distributed between the superior sagittal sinus and periosteal layer of the dura mater [27].

However, despite these studies showing immunoreactive meningeal lymphatic vessels, the available literature about lymphatic vessels in humans is not consistent. It seems that when working with autopsy material, a protocol for antigen retrieval is essential in order to do immunostaining of meningeal lymphatic vessels, as more recently demonstrated by Mezey et al. [26]. We were, however, not able to label lymphatic vessels in this way, despite having tried various methods of antigen retrieval. In contrast, using the freezing method, we were able to obtain good staining consistently without the need of antigen retrieval. The discrepancies may stem from variations in the fixation and autopsy procedures performed. Supporting this idea, Johnson [28] examined dura mater of 113 autopsy specimens and reported microscopic fluid channels in 101 of 113 (90%) of the dura specimens; they were on the meningeal side of dura, variable in size, and podoplanin-negative. They observed D2-40 expression, indicative of lymphatic vessels, in only seven autopsies. Interestingly, the lymphatic structures were either linear or organized in clusters, either nearby or remote of blood vessels. More recently, Park et al. [38] in nine autopsy specimens failed to demonstrate D2-40 immunoreactive lymphatic vessels in human dura, and reported a network of dural channels within the parasagittal dura not in association with lymphatic vessels. Based on our present observations, it may be speculated whether the absence or low frequency of immunoreactive lymphatic vessels in these two latter studies was due to the fixation procedure of the autopsy specimens. Alternatively, the microscopic dural channels are non-lymphoid structures, which may compare with the observation that the fluid channels are smaller than the previously reported dural lymphatic vessels.

The present data unequivocally demonstrate that the fixation method is essential for the ability to demonstrate meningeal vessels in human dura. Evidently, fixation with PFA 4.0% is too strong and since the dimension of the sample is small, it most likely masks the epitopes in the LYVE-1 receptor and therefore prevents binding of antibodies. The antigen might as well become denatured by PFA 4%. This may be a bit surprising given the common clinical practice to demonstrate extra-cranial lymphatic vessels utilizing the antibody D2-40 [39, 40]. That begs the question whether lymphatic endothelium in the CNS exhibits surface protein isoforms that are more sensitive to fixation procedures than their counterparts in the rest of the body and if such were the case, what functional implications would this have for lymphatic vessels in the meninges?

The present study only included patients with iNPH since we here primarily addressed methodological aspects of immunohistochemistry of meningeal lymphatic vessels. Future studies are needed to clarify whether meningeal lymphatic function is abnormal in iNPH, and whether this is reflected in smaller diameter of the meningeal lymphatic vessels. It is worthy to note that iNPH overlaps with Alzheimer’s regarding deposition of amyloid-β within the brain [41]. In this regard, impaired meningeal clearance of amyloid-β might hypothetically be a key factor in iNPH. The present data is not suitable for deciphering the role of meningeal lymphatic function in iNPH, but if meningeal lymphatic dysfunction is a key factor in iNPH pathogenesis, an interesting approach would be lymphatic function enhancement, e.g. utilizing VEGFR3-specific recombinant VEGF-c [20]. Likewise, iNPH is a disease of the elderly and it has been shown that meningeal lymphatic function declines with increasing age. For these reasons, dura biopsies during shunt surgery might provide diagnostic information in this disease.

One interesting question is whether the dimensions of meningeal lymphatic vessels in iNPH are smaller than in healthy subjects. The diameter of meningeal lymphatic vessels previously reported in humans vary, and were ranging between 19 and 470 μm in one study [27], and 7 to 842 μm (average ± stdev: 125 ± 161 μm) in another study [23]. In comparison, the diameter of rodent meningeal lymphatic vessels was in the range 20 to 30 μm [17, 18]. Considering lymphatic vessels both nearby and remote from blood vessels in the present study, the total width of lymphatic vessels was 46.7 ± 24.0 μm (ranges 10.1 to 87.7 μm) with wall thickness 6.6 ± 4.3 μm (ranges 1.7 to 17.1 μm) and lumen diameter 27.4 ± 15.5 μm (ranges 6.7 to 66.2 μm). Accordingly, the dimensions of lymphatic vessels in the present study are smaller than previously reported. It could be argued that the reason behind this is the location of the tissue examined and that lymphatic vessels vary greatly in their size, relative to the distance of the superior sagittal sinus. Lymphatic vessels in the rest of the body vary as well. For example, initial lymphatic capillaries have an approximate diameter of 10–60 μm [42]. Therefore, it is possible that the lymphatic vessels observed in the present study are small collecting capillaries that transport their content into larger lymphatic vessels closer to the superior sagittal sinus. If so, this might suggest that lymphatic vessels become smaller in caliber as they move laterally from the superior sagittal sinus.

We cannot decipher from the present data the function of meningeal lymphatic vessels in humans, but the literature is growing concerning the putative role of meningeal lymphatic vessels for normal brain function. An increasing body of evidence points at the role of meningeal lymphatic vessels in immune surveillance [43]. In particular, the more recent observations of passage of fluid and cells between CSF, dura and skull bone marrow [44–46] are intriguing as they provide for immunological peripheral-central cross talk at the meninges, both under physiological and pathological conditions. Moreover, the evidence is growing that meningeal lymphatic vessels serve a function in clearing CSF and solutes [19], including metabolic waste products such as soluble amyloid-β [20], tau [21] and α-synuclein [22]. Hence, impaired or abolished glymphatic/meningeal lymphatic waste clearance of the waste products may play a role in the pathophysiology of Alzheimer’s (soluble amyloid-β and tau) and Parkinson’s diseases (α-synuclein) [47].

Some limitations with the present study should be noted. The statistical comparisons between groups was hampered by a rather low number of cases for Methods #1 and #2. Furthermore, the distance from the superior sagittal sinus differed a few millimeters between the groups (Table 2). We do not think this would affect the results as the dura samples were obtained from the same location in all subjects. On the other hand, this also makes it impossible to conclude about variability in expression of lymphatic vessels throughout the dura. We here used the same location for the dura sample, as the aim of this study was to compare the effect of different tissue fixation protocols and their effect on antigenicity for immunohistochemistry. For that purpose, sampling from the same region was optimal. The present dura specimens were obtained from iNPH patients, making it impossible to conclude about meningeal lymphatic vessels in healthy subjects. In addition, we are not able to distinguish between CD45 + LYVE1 + macrophages and CD45- LYVE1 + lymphatic endothelial cells without the aforementioned marker, making it hard to make conclusions about the abundance of lymphatic meningeal vessels in these subjects. Moreover, we cannot conclude whether lymphangiogenesis is up- or down-regulated in iNPH patients. Further studies are required to determine how the distribution of meningeal lymphatic vessels differs throughout the dura in humans. Are they primarily distributed along the dural sinuses? An interesting point is whether the density of lymphatic vessels might differ between different disease categories. Lastly, due to the fixation method in order to preserve as much LYVE-1 antigenicity as possible, lymphatic vessel staining between adjacent sections was highly variable and in some cases the full extension of a lymphatic vessels’ surface in a single section was not labelled completely making it difficult to do systematic measurements and stereology was not possible to perform. Therefore, the measurements done in the present study were taken only from the sections where the vast majority a lymphatic vessel was visualized, which might introduce errors in width/lumen as the 3D structure changes throughout the tissue.

## Conclusions

The present observations show that visualization of meningeal vessels in human dura is highly sensitive to the fixation method. We here for the first time demonstrate meningeal lymphatic vessels in patients, and show that human meningeal lymphatic vessels are located both in intimate vicinity to blood vessels as well as remote, and LYVE-1-expressing cells were organized in clusters. The present observations open for further studies on the characteristics and implications of human meningeal lymphatic vessels.

## Electronic supplementary material

Below is the link to the electronic supplementary material.

Supplementary movie 1. Meningeal lymphatic vessels in close proximity to blood vessels. Lymphatic vessels are visualized in green color and blood vessels in red color. See Fig. 4D.

Supplementary movie 2. Meningeal lymphatic vessels in distance from blood vessels. Lymphatic vessels are visualized in green color. See Fig. 5D.

Supplementary movie 3. Cluster of LYVE-1-expressing cells. Cluster of LYVE-1-expressing cells are visualized in green color. See Fig. 6D.

Supplementary Material 4

## Data Availability

The data that support the findings of this study are available from the corresponding author, upon reasonable request.

## List of abbreviations

CCL21: Chemokine ligand 21
CNS: Central nervous system
CSF: Cerebrospinal fluid
IHC: Immunohistochemistry
iNPH: Idiopathic normal pressure hydrocephalus.
LEC: Lymphatic endothelial cell
LYVE-1: Lymphatic vessel endothelial hyaluronan receptor 1
MLV: Meningeal lymphatic vessels.
MRI: Magnetic resonance imaging
PFA: Paraformaldehyde
PDPN: Podoplanin
T2-FLAIR: T2- weighted fluid-attenuation inversion recovery
VEGFR-3: Vascular endothelial growth factor 3
VP: Ventriculoperitoneal
