# Supplementary material for: Immunohistochemical visualization of lymphatic vessels in human dura mater: methodological perspectives
Source: Fluids Barriers CNS. 2023 Mar 28;20:23. doi: 10.1186/s12987-023-00426-3 (PMC10044429; doi:10.1186/s12987-023-00426-3)
Supplement: Supplementary file 4 — Supplementary Material 4 [file 12987_2023_426_MOESM4_ESM.pdf]

Supplementary Material

Immunohistochemical visualization of lymphatic vessels in human dura mater: Methodological perspectives

César Luis Vera Quesada, MD, MSc[1,2] Shreyas Balachandra Rao, MSc, PhD,[3] Reidun Torp, MSc, PhD[3] Per Kristian Eide, MD, PhD[1,2]

[1]Department of Neurosurgery, Oslo University Hospital-Rikshospitalet, Oslo, Norway

[2]Institute of Clinical Medicine, Faculty of Medicine, University of Oslo, Oslo, Norway,

[3]Division of Anatomy, Department of Molecular Medicine, Institute of Basic Medical Sciences, University of Oslo, Oslo, Norway

Corresponding author:

Professor Per Kristian Eide, MD PhD

Dept. of Neurosurgery,

Oslo University Hospital – Rikshospitalet,

PB 4950 Nydalen, 0424 OSLO, Norway

Phone: +47 91649419; Fax: +47-23074310

E-mail: p.k.eide@medisin.uio.no

Supplementary Figure 1

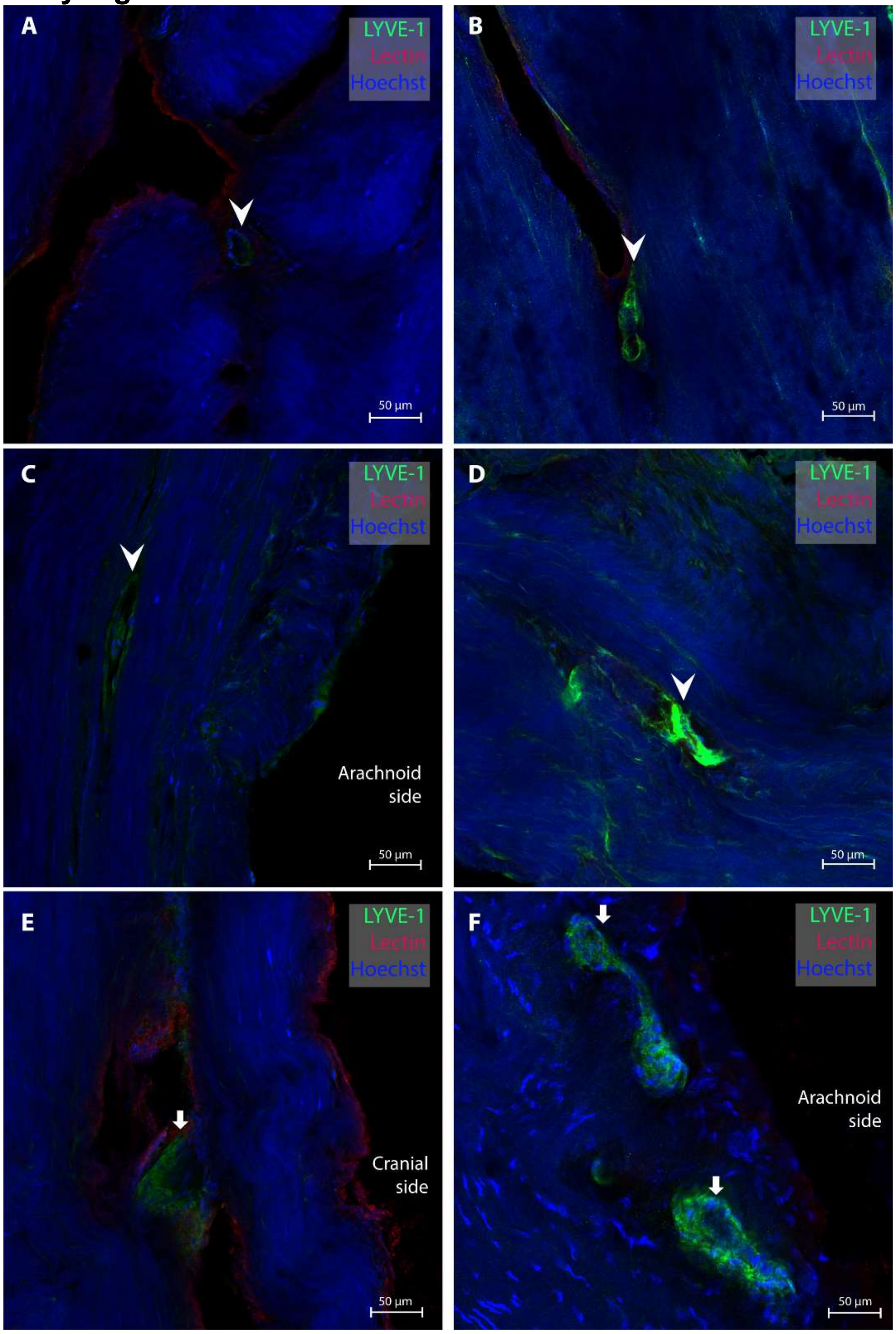

Comparison between fixation with 0.5%PFA and freezing method. The sections on the left column (**A**, **C**, **E**) were fixed in 0.5% PFA overnight while the sections on the right column (**B**, **D**, **F**) were frozen immediately after dissection and fixed 10 minutes in 0.5% PFA before IHC. Note the difference in signal intensity between fixation protocols. Top panels (**A** patient #27, **B** patient #21) are lymphatic vessels (white arrowheads) in proximity to blood vessels, middle panels (**C** patient #28, **D** patient #22) are lymphatic vessels (white arrowheads) in distance to blood vessels and bottom panel (**E** patient #26, **F** patient #13) are “clusters” (white arrows) of lymphatic tissue, some of which may be collapsed lymphatic vessels.

Supplementary Figure 2

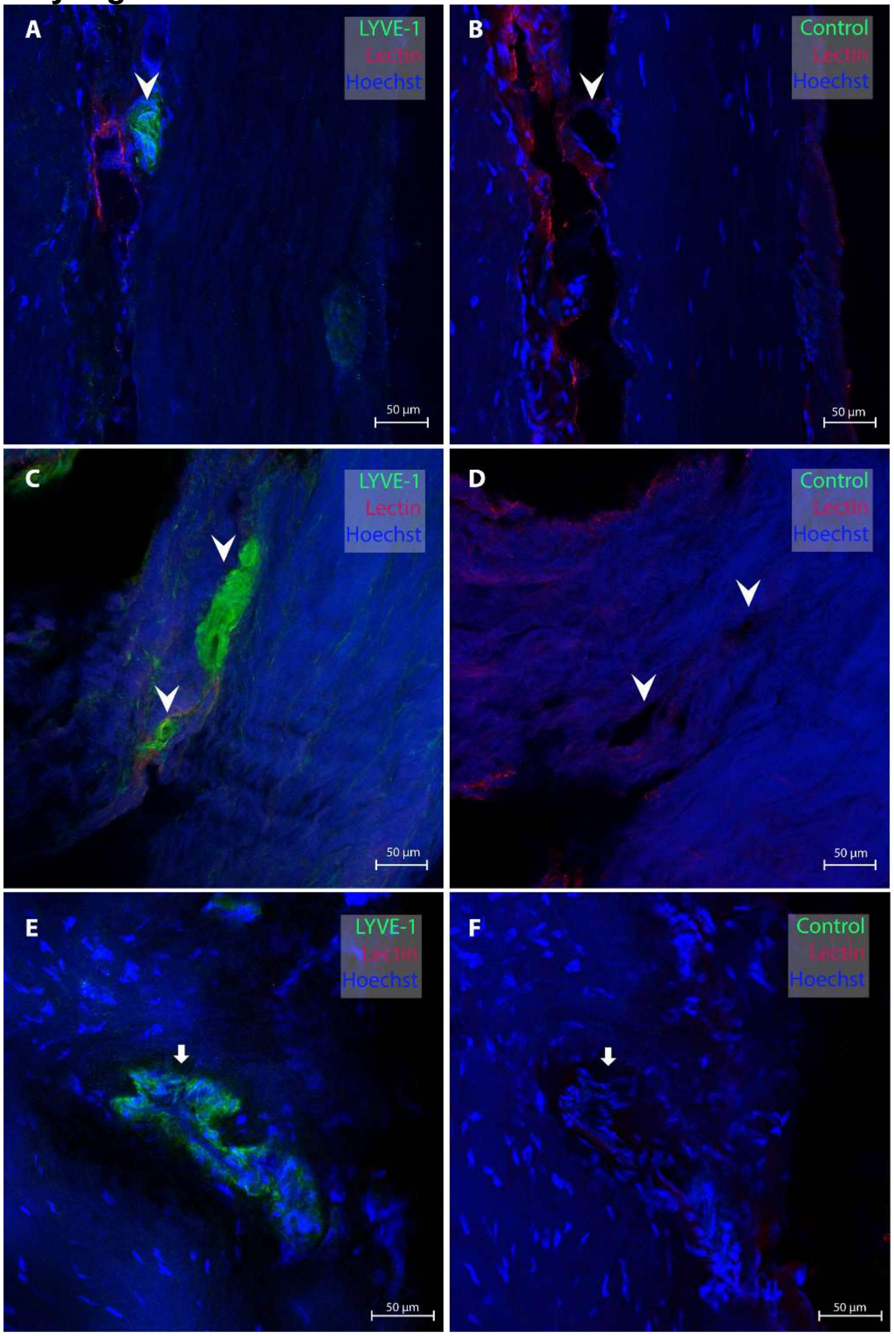

Control experiments showing specific antibody binding to lymphatic structures. For sections on the left column (**A**, **C**, **E**) primary anti-LYVE-1 antibody and secondary CY3 antibody was used for staining. Sections on the right column (**B**, **D**, **F**) were immediately adjacent to sections on the left used as controls where no primary antibody against LYVE-1was used. Top panels (**A**, **B** from patient #8) are lymphatic vessels (white arrowheads) in proximity to blood vessels, middle panels (**C**, **D** from patient #9) are lymphatic vessels (white arrowheads) in distance to blood vessels and bottom panel (**E**, **F** from patient #13) are “clusters” (white arrows) of lymphatic tissue.

Supplementary Figure 3

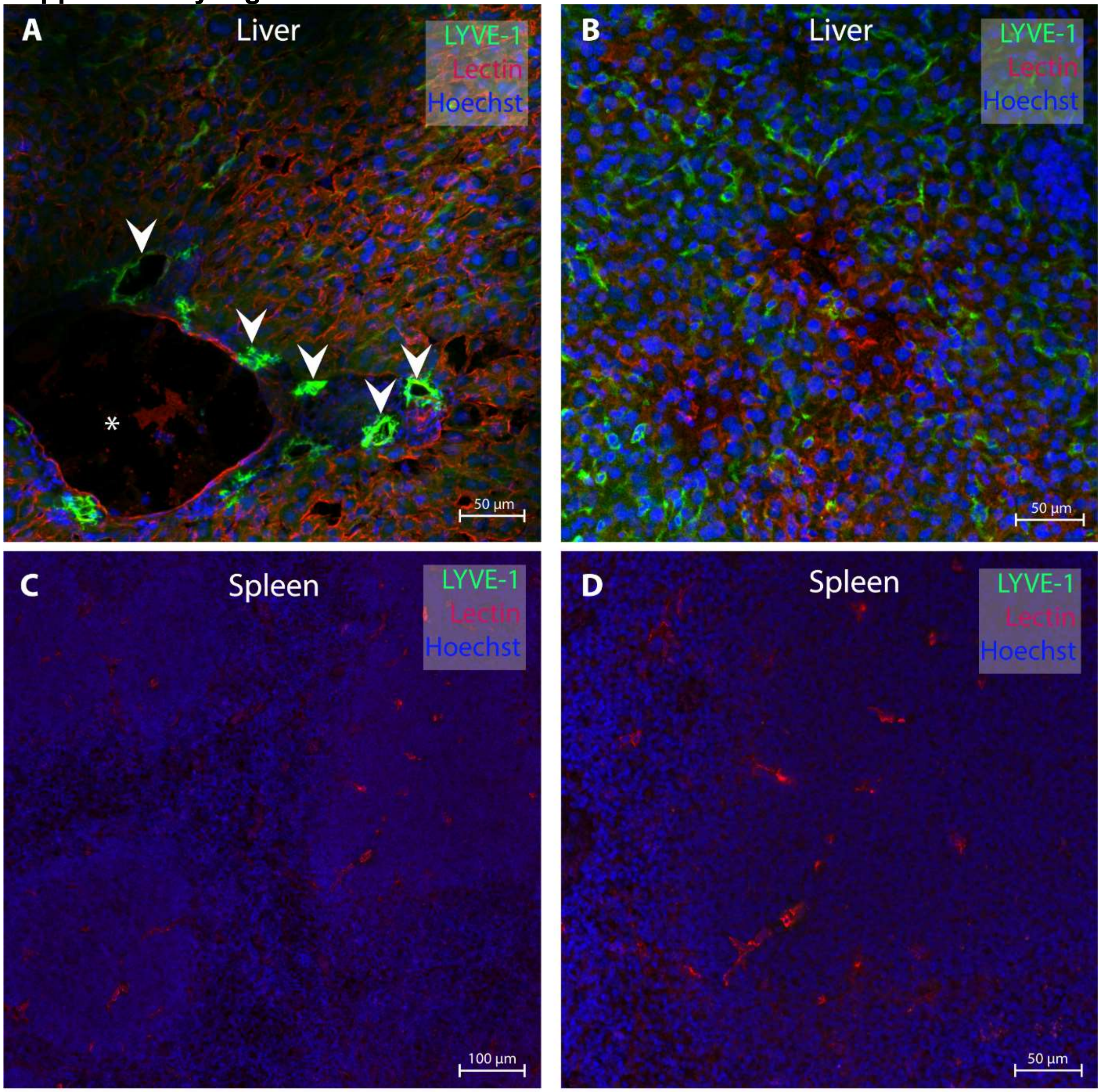

Positive and negative controls of LYVE-1 staining in mouse tissue. Top panels (**A**, **B**) are mouse liver sections (positive control) showing stained lymphatic vessels (white arrowheads) and blood vessels (asterisk) using antibodies against LYVE-1 and Lectin. Bottom panels (**C**, **D**) are mouse spleen sections showing absence of LYVE-1 as expected (negative controls).

Supplementary Figure 4

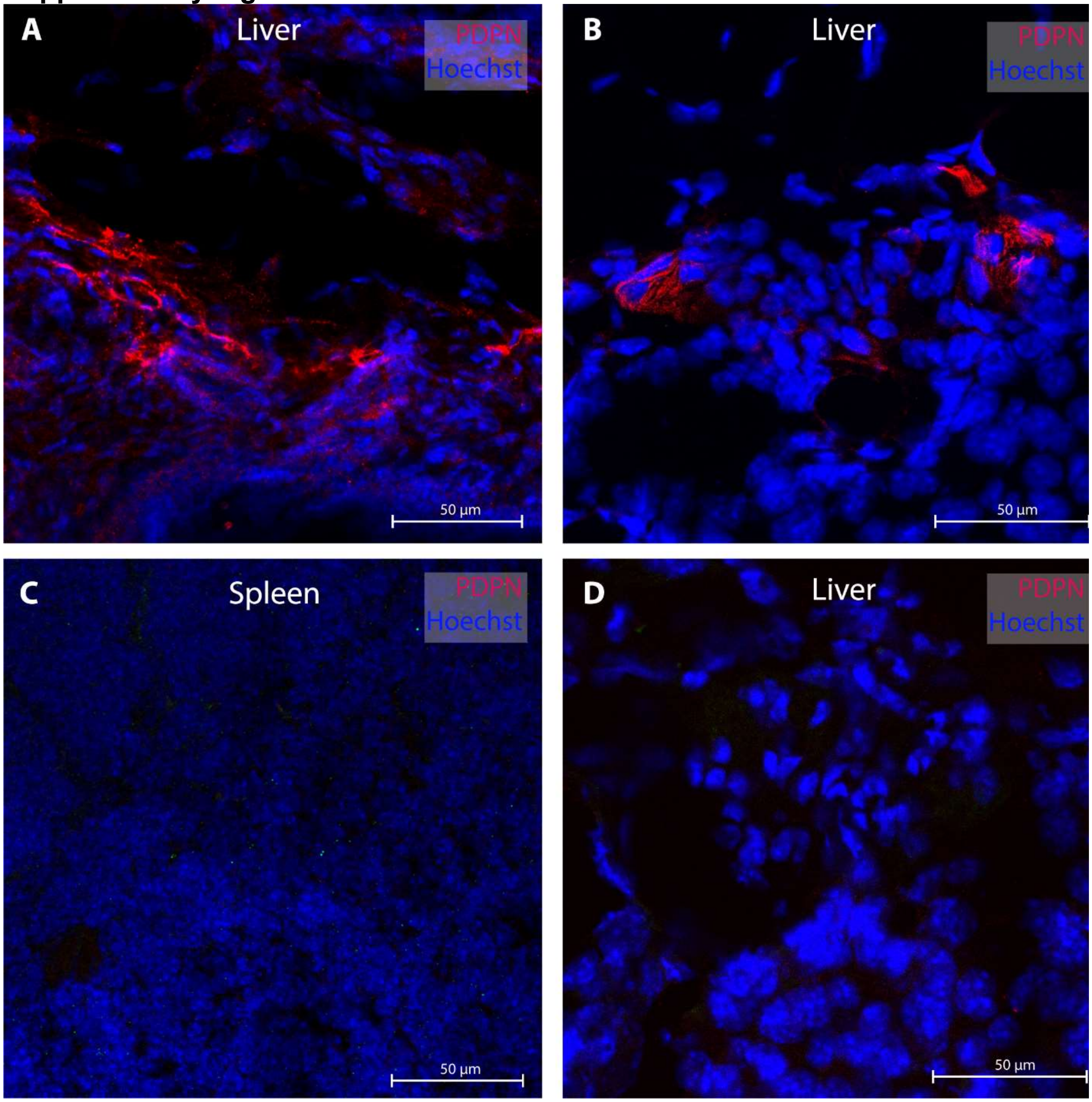

Positive and negative controls of PDPN staining in mouse tissue. Top panels (**A**, **B**) are mouse liver sections (positive control) showing stained lymphatic vessels using antibodies against PDPN. Bottom panel **C** is a mouse spleen section showing absence of PDPN as negative control while panel **D** is a serial section of panel **B** used as negative control where no primary antibody was added.

Supplementary Figure 5

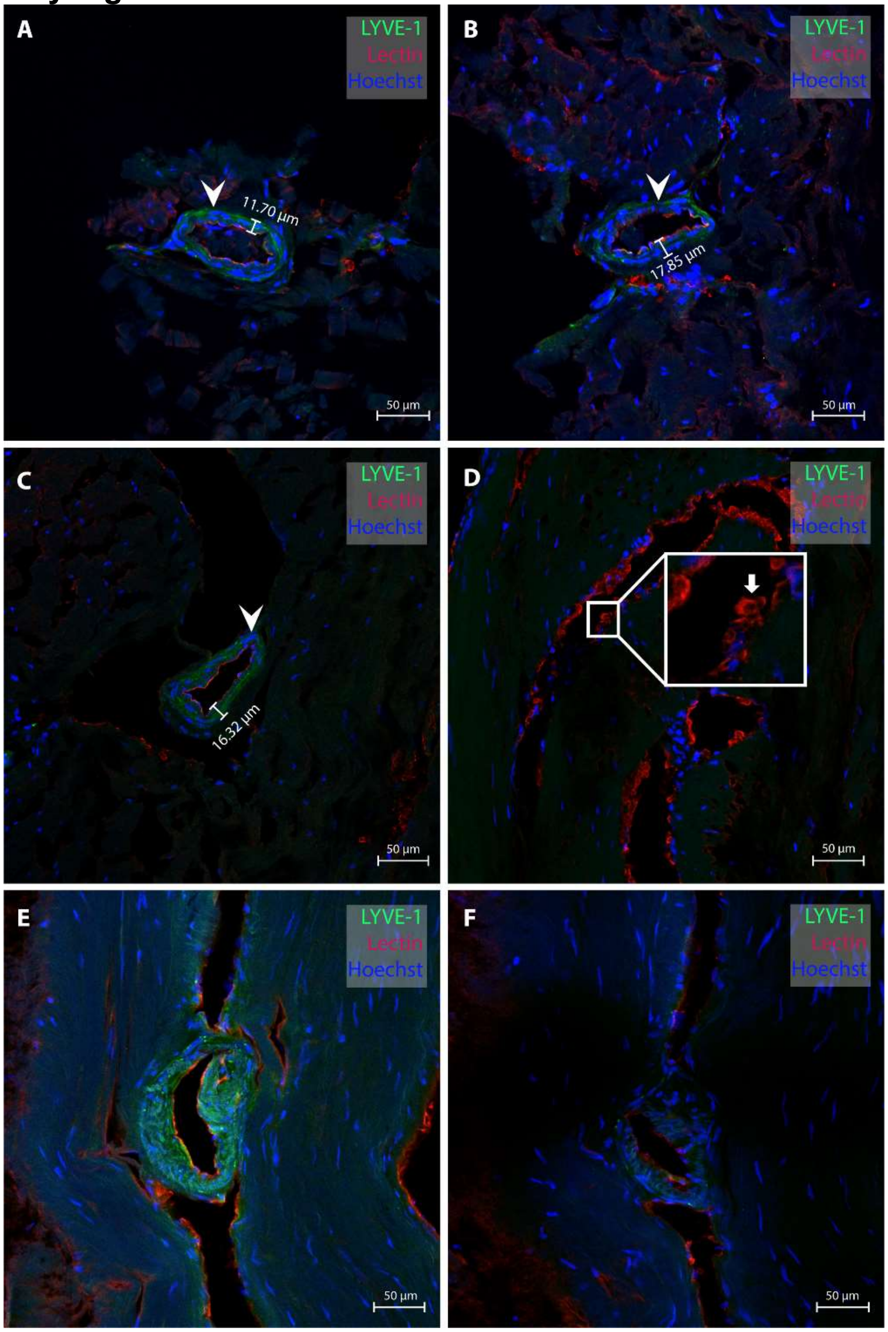

Separating lymphatic vessels from blood vessels. A-C (patient #6). Blood vessels (white arrowheads) express LYVE-1 in their tunica media, but with vascular endothelial cells expressing Lectin (red). Blood vessel walls are generally thicker (A: 11.70 µm, B: 17.85 µm, C: 16.32 µm) compared to lymphatic vessels. Panel **D** (patient #5) shows labelled erythrocytes (white arrow) within lumen of blood vessel. Panel **E** (patient #2) shows positive LYVE staining with lectin stained endothelium. Panel **F** shows the same vessel in another section as negative control, as illustrated in Figure 8, where no primary anti-LYVE-1 antibody was added during immunohistochemistry disproving background staining.
